# Epigenome-Wide Association Studies (EWAS) in Cancer

**DOI:** 10.2174/138920212800793294

**Published:** 2012-06

**Authors:** Mukesh Verma

**Affiliations:** Epidemiology and Genetics Research Program, Division of Cancer Control and Population Sciences, Division of Epidemiology and Genetics, National Cancer Institute, National Institutes of Health (NIH), 6130 Executive Boulevard, Suite 5100, Bethesda, MD 20892-7324, USA

**Keywords:** Acetylation, biomarker, chromatin, environmental mutagens, epidemiology, epigenetics, histone acetyl transferase (HAT), histone, histone deacetylase (HDAC), histone code, imprinting, methylation, methyl transferase, mutagens, risk assessment, screening.

## Abstract

After completion of the human genome, genome-wide association studies were conducted to identify single nucleotide polymorphisms (SNPs) associated with cancer initiation and progression. Most of the studies identified SNPs that were located outside the coding region, and the odds ratios were too low to implement in clinical practice. Although the genome gives information about genome sequence and structure, the human epigenome provides functional aspects of the genome. Epigenome-wide association studies (EWAS) provide an opportunity to identify genome-wide epigenetic variants that are associated with cancer. However, there are problems and issues in implementing EWAS to establish an association between epigenetic profiles and cancer. Few challenges include selection and handling of samples, choice of population and sample size, accurate measurement of exposure, integrating data, and insufficient information about the role of repeat sequences. The current status of EWAS, challenges in the field, and their potential solutions are discussed in this article.

## INTRODUCTION

Biology in general has been genocentric for decades, although the fate of a gene is not defined by the DNA sequence but by how a gene is programmed by chromatin changes, DNA methylation, and noncoding RNAs. Epigenetics defines mechanisms that involve mitotically heritable changes in DNA and chromatin that affect gene expression without altering the nucleotide sequence [1, 2]. Therefore, the functional importance of epigenetic changes lies in their ability to regulate gene expression. One of the current challenges is to understand the regulation of gene function, an activity that depends largely on epigenetic control. Four major steps in epigenetic regulation are promoter methylation, histone acetylation/deacetylation, noncoding mRNA expression, and chromatin conformational changes [3, 4]. Through their effects on chromatin structure, epigenetic changes can modulate transcriptional repression, X-chromosome inactivation, genomic imprinting, and suppression of the detrimental effects of repetitive and parasitic DNA sequences on genome integrity [5, 6]. Following the completion of genome-wide association studies (GWAS) in several cancers, it was observed that disease-associated single nucleotide polymorphisms (SNPs) are not localized near any gene in the pathways involved in these cancers. Perhaps now is the right time to evaluate the status of alternative approaches such as epigenome-wide association studies (EWAS) to determine whether EWAS have evolved to the extent that they can be useful in identifying disease-associated marks for screening high-risk populations and in developing strategies for cancer control and treatment. Or, are there issues concerning EWAS approaches that should be resolved before moving in that direction?

## BACKGROUND: KEY COMPONENTS OF THE EPIGENOME

Methylated DNA is a key component of epigenetic information in mammalian and other cells. DNA methylation involves the covalent addition of a methyl group to the C5 position of cytosine within CpG dinucleotides, which often are clustered as CpG islands in the promoter regions of genes [7]. DNA methylation also can occur in other parts of genes. Because DNA methyl transfer reactions occur on one strand at a time, *de novo* methylation leading to full double-stranded DNA methylation can be thought of as two sequential one-stranded reactions. Recent work has revealed that DNA methylation plays a key role in DNA repair, genome instability, and regulation of chromatin structure [8]. 

Epigenetic control mechanisms can become dysregulated in cancer cells. Such dysregulation can affect a variety of gene types, including tumor suppressor genes, oncogenes, and cancer-associated viral genes. Prevailing evidence suggests that epigenetic changes, especially DNA methylation, may play a role in cancer etiology. Genomic methylation patterns frequently are altered in tumor cells, with global hypomethylation accompanying region-specific hypermethylation events. When hypermethylation occurs within the promoter of a tumor suppressor gene, expression of the associated gene can be silenced.

Another focus of the scientific investigation of epigenetic regulation involves histone acetylation and deacetylation. Histones are proteins that bind to the DNA to neutralize the basic charge of the DNA in chromatin. Histone deacetylation leads to chromatin condensation, with concomitant transcriptional repression. Conversely, histone acetylation, which involves the covalent addition of acetyl groups to the lysine moieties in the *N*-terminal histone tails, appears to result in the decondensation of chromatin, which is associated with upregulation of gene expression [6]. Histone acetylation and deacetylation function in a dynamic equilibrium in a manner that is regulated by histone acetyltransferases (HATs) and histone deacetylases (HDACs) [9]. Exogenous agents can alter the quantitative balance between HATs and HDACs and, thus, the dynamics of histone acetylation/deacetylation [10]. Agents that alter net acetylation, thereby affecting chromatin decondensation and gene expression, are only effective in the context of previously “competent” chromatin (i.e., partially transcriptionally active chromatin). It has been discovered that non-coding RNAs (ncRNAs) play a significant role in epigenetics mediated gene regulation. The most studied ncRNAs are short microRNAs (miRNAs), PIWI- interacting RNAs (piRNAs) and large intergenic non-coding RNAs (lincRNAs). These ncRNAs can self-propagate and be transmitted independently of the underlying DNA. Thus, ncRNAs can epignetically transmit regulatory information. To study epigenetic regulation completely and to minimize variations, all components of epigenetic regulation (methylation, histone modification, and noncoding RNA expression) should be studied in the same sample. 

### Environment and the Epigenome

Alterations in DNA methylation, histones, and miRNA in response to environmental exposures demand a new generation of exposure biomarkers [11]. A variety of chemicals (such as nickel, arsenic, cadmium), certain base analogs, radiation, smoke, stress, hormones (such as estradiol), infectious agents, and reactive oxygen and nitrogen species can alter the phenotypes of mammalian cells, *via *epigenetic mechanisms, without changing the underlying DNA sequence. These agents can alter the methylation and/or acetylation state of the DNA. Another context in which DNA methylation exerts important control over gene expression involves viral genes. Methylation of the promoter region of the viral genome maintains the latency of the virus, thereby preventing the expression of viral antigens. In this article, I use “environment” in the broader sense of lifestyle, infections, radiation, natural and man-made chemicals, and occupational exposures. In lung cancer, for example, there is a strong link between the environment (smoking, radon exposure, asbestos) and disease, whereas in other cancers the environment interacts with a gene to exert its effect and contribute to disease development.

The primary genome sequence generally is static, but epigenetic marks are transient and modifiable (by the environment) throughout their life. Epigenetic mechanisms allow responding to the environment through changes in gene expression. Evidence linking environmental changes and the epigenomic state of the genome is growing rapidly [12, 13]. 

Unlike genetic changes, epigenetic alterations can be reversed chemically [14]. The reversal of epigenetic alterations has implications for cancer treatment. To date, four epigenetic inhibitors have been approved by the U.S. Food and Drug Administration (FDA) for cancer treatment.

### Why EWAS?

Despite intriguing leads that have resulted from epigenetic studies of cancer, most human studies of epigenetics are too small, focus on small genomic regions, and study only one component of the epigenome. To understand epigenetic regulation completely and to reduce sample-related variability, all components of epigenetic regulation (methylation, histone modification, and miRNA expression) should be studied in the same sample. In addition, GWAS in human populations have been successful in identifying previously unidentified genomic regions that are associated with the risk of certain cancers, validated findings for some cancers in regions associated with previously identified candidate genes, and helped to explain some racial disparities in cancer incidence. GWAS examine genetic variants in the germlines of persons with and without cancer or in persons with cancer who experience different cancer outcomes. It seems likely that a similar agnostic approach to querying the epigenome might meet with similar success. However, GWAS findings have limitations, including that relative risks are modest. To date, GWAS of some cancers, such as breast and prostate cancers, have failed to identify any risk-associated SNPs that are located in the coding regions of genes known to play a role in the development of these cancers. It will be important to correlate findings from EWAS with existing GWAS findings. EWAS may provide functional correlation of genes associated with the risk of cancer. Technologies are available to profile methylation, histone modifications, and miRNAs [3, 15-17]; however, epidemiologic studies have not been conducted using histone profiles, and miRNA profiles have been used in a limited number of studies. An ideal epidemiologic study must include thousands of study participants to address the problem of false-positive findings due to multiple comparisons being made. The costs of simultaneously conducting large-scale methylation profiles across the genome and of fully characterizing histone modifications and miRNAs should be reasonable, making it possible to use these technologies in studies that involve many thousands of cases and appropriate controls or cancer cases with different outcomes [4, 18, 19]. All components of the epigenome can be assessed in the relatively noninvasively collected biospecimens that typically are available in the large cancer epidemiology studies that seek to identify risk factors for cancer, cancer recurrence, or survival after cancer [20-23]. By conducting EWAS, we can collect information about epigenomic variations throughout the genome/epigenome. 

The majority of work in epigenetics and epidemiology has been completed in the field of methylation. Methylation assays are DNA based, and the starting material is very stable. However, histone assays are antibody based, and miRNA assays are RNA based; both of which require expertise in sample handling. The National Cancer Institute (NCI) has initiated The Cancer Genome Atlas (TCGA) program to characterize high-quality biospecimens at the genetic and epigenetic levels (http://cancergenome.nih.gov/). At the National Institutes of Health (NIH), the complete human epigenome is being characterized. This will serve as a reference to identify disease-associated marks (http://commonfund.nih.gov/epigenomics/referenceepigenomeconsortium.aspx).

Another positive aspect of epigenome studies is their therapeutic potential. Reagents have been discovered that, when used either alone or in combination, are effective anticancer agents [24]. For example, oridonin, a tetracycline diterpenoid compound, has been effective against colorectal cancer. The mechanisms involved are the induction of histone (H3 and H4) hyperacetylation; activation of *p21, p27,* and *p16*; and suppression of *c-myc* expression [24].

## CURRENT EWAS STUDIES: EXAMPLES OF GENOME-WIDE ANALYSIS OF EPIGENETIC COMPONENTS

There are no examples in which all four of the components of epigenetics have been studied at the genome-wide level. In the following section, I discuss examples of methylation and miRNA profiling studies. 

### Genome-Wide Methylation Analysis in Prostate Cancer

Jong Park of the Moffitt Cancer Center is interested in identifying epigenetic events involved in the recurrence of prostate cancer [25]. He is using samples from an ongoing cohort in which prostate cancer patients were treated with radiation. To date, researchers have not identified genetic and epigenetic markers that can help distinguish between prostate cancer patients who should undergo extensive therapy and those who should have observation without treatment. Identifying markers of the recurrence of prostate cancer is an urgent need. Such studies may have potential for understanding pharmacoepigenomics.

### Genome-Wide Methylation Analysis in Head and Neck Cancer

While studying head and neck squamous cell carcinoma (HNSCC), investigators at the University of Cincinnati are interested in identifying epigenetic and genetic factors that contribute to HNSCC development in highly sensitive (HS) and highly resistant (HR) smokers in a cohort of smokers and nonsmokers with more than 4,000 participants [26-29]. The focus of this research is on identifying genetic factors (by GWAS and copy number variance profile), methylation profiles, and urinary metabolite profiles in 500–1,000 HR and HS smokers for a period of 4 years. Integration of the findings from these studies will be achieved by performing conditional statistical analyses and pathway analyses and then validating the important genetic networks and metabolic pathways by studying tumor tissues in cell culture. Identification of epigenetic and genetic variations by these methodologies will enhance understanding of the mechanisms of susceptibility to HNSCC. Many heavy smokers do not develop HNSCC, whereas 10–15% of patients with little or no exposure to cigarettes do develop HNSCC. This clearly suggests an underlying epigenetic predisposition to this type of malignancy. Nebert and colleagues applied an extreme discordant phenotype (EDP) method of analysis, and the statistical power of this method in specific cases of a not-so-rare trait of HNSCC and environmental exposure is well documented [26-30].

### Genome-Wide Histone Modifications in Gliomas

Gliomas start in the brain or spine, and very few markers are available to detect this tumor. Liu *et al*. [31] studied global histone modification patterns in 230 tumor samples from patients and identified specific histone modification profiles as prognostic markers. Alterations in histone 3 lysine 4 dimethylation (H3K4diMe); histone 4 arginine 3 monomethylation (H4R3monoMe); histone 4 lysine 20 trimethylation (H4K20triMe); and acetylation of histone 3 lysine 9 (H3K9Ac), histone 3 lysine 18 (H3K18Ac), histone 4 lysine 12 (H4K12Ac), and histone 4 lysine 16 (H4K16Ac) were reported. In another study, genome-wide histone mapping was completed in type 2 diabetes [32]. The main advantage of such studies lies in translational research because histone profiles will guide prognosis and provide optimal adjuvant treatment options. Furthermore, such studies provide a rationale for using novel HDAC inhibitors instead of regular chemotherapeutic agents. Molecular signatures identified in cancer also may help in detecting residual disease after initial treatment. The potential to distinguish pre-invasive from invasive diseases also exists if disease-associated markers are identified and validated.

A basic question to be considered is what are the epigenetic events that play a significant role in cancer initiation, progression, development, and recurrence; and what are the environmental factors that affect those events? Further research is needed to answer this question.

## RESEARCH DESIGN, TECHNOLOGIES, AND STATISTICAL ANALYSIS IN EWAS

Different types of high-quality samples in large numbers are suitable for EWAS (Table **1**). Most of the reports are on blood samples, but tissue samples and other biofluids also have been used extensively. For example, esophageal cancer methylation profiling was conducted on fresh-frozen tissue specimens (tumor, low- and high-grade dysplasia) [33]. This group has followed a temporal epigenome program mapping strategy. Samples are collected from advanced stages of Barrett’s metaplasia with known outcomes. Two categories, progressors and nonprogressors, then are selected. Progressors are cases that have reached a high-grade dysplasia or esophageal adenocarcinoma (EAC); nonprogressors are cases that did not progress to EAC. Samples are collected at different time intervals, and their methylation profiles are determined. A methylation-sensitive restriction-enzyme-based method is applied to determine the profile. To perform two-way comparisons, a Student’s t-test (parametric testing) and Mann-Whitney U-test (nonparametric testing) are applied to the log and nonlog versions of the methylation array data. Further analysis is conducted to construct classifiers to distinguish study Category I from Category II (*e.g., *progressors from nonprogressors) based on these methylation array data. Classifier methods include prediction analysis of microarrays (PAM) and artificial neural networks (ANNs). Outliers are identified using these methods. Sometimes, unsupervised methods such as hierarchical clustering also are utilized to look for natural groupings in the specimens and among the methylation loci [34].

## CHALLENGES AND OPPORTUNITIES

**Fig. (1):**
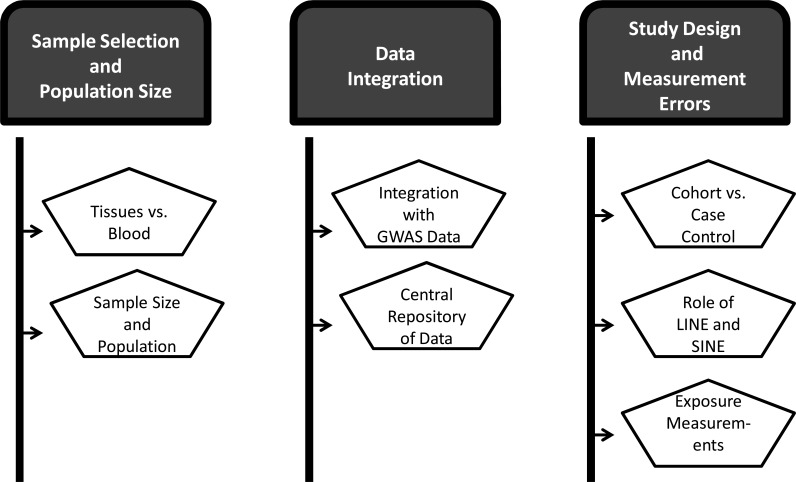
Challenges in EWAS.

A number of challenges arise when conducting EWAS (Fig. **1**). I discuss different categories of these challenges below.

The main challenge in the EWAS field is the selection of samples. In epidemiologic studies, blood is the most common sample source. In ideal situations, tissues should be used for any analysis because epigenetic signatures are different in different types of cells. However, tissue collection involves invasive technologies, and problems arise when samples are collected from the same subjects for follow-up studies. 

Populations are selected for epidemiologic studies based on the research questions. Problems associated with low recruitment and dropout rates should be considered before planning a study with a large number of subjects. Reaching an adequate high-quality sample size remains a challenge in epidemiologic studies. Different kinds of biological samples have been used for epigenetic analysis (see Table **1**). Variations between persons and within a person may be challenging when considering the dynamic nature of epigenetic-mediated gene regulation. Unlike genomic information, which is static, epigenomic information changes with time. 

Epigenetic profiles respond to environmental changes, and identifying disease-specific profiles requires knowledge of exposures, modifiable factors, and their effects. Exposure measurement limitations and the subsequent risk of misclassification present another major challenge. Improved exposure assessment techniques are needed. Several papers assess the contribution of environmental factors in cancer etiology at 1 to 19% [35-40]. Alterations in epigenetic profiles may be due to specific genes or to a large section of the genome. The effects of gene-environment interactions already are taken into account because the epigenotype reflects the effects of the environment and genes on the epigenome. Folic acid, choline, and methionine are known to directly affect epigenetic profiles [41-43].

After epigenetic profiles and markers are identified, they should be mapped, and their relationship should be established in large-scale studies. The large amount of data produced from each run (may exceed 1Gb) poses another challenge for EWAS. Thus far, the genome-wide epigenetic profiling of LINE and SINE sequencing has not been completed. When these data are combined with previously collected data, it will be challenging to integrate all of these data and reach useful conclusions. Only a few laboratories have the expertise required to conduct analyses of all four of the components of epigenetics. Collaboration is needed among investigators with different expertise, such as in methylation, histone, chromatin, miRNA, epidemiology, bioinformatics, and statistics. Partnerships with industry may be helpful in this area. Histone profiling is based on ChIP-on-chip analysis, for which high-quality monoclonal antibodies should be used. Few commercially available monoclonal antibodies are of high quality, however. The NIH Epigenome Roadmap has initiated a program to generate monoclonal antibodies against all major histone modifications (http://commonfund.nih.gov/epigenomics/index.asp). These antibodies will be made readily available to investigators. 

New tools are needed to fully utilize GWAS data and determine their correlation with EWAS data. These tools should facilitate the integration of genetic and environmental exposure data into EWAS data to enable the identification of populations at high risk of developing cancer. Data generated by the NIH Epigenome Roadmap program are maintained at the National Center for Bioinformatics Information (NCBI) and dbGAP (the Database of Genotypes and Phenotypes). GWAS data are stored and maintained at dbGAP. Currently, however, no arrangements have been made for storing and maintaining EWAS data at a central location. A variety of approaches have been adopted for epigenetic and epidemiologic studies, such as cohort studies, case-control studies, cross-sectional studies, intervention studies, family-based studies, twin cohorts, and birth cohorts, but the best approach has not been determined. High-throughput technologies are required for these studies. Thus far, most studies have been completed using differential methylation profiles.

Much of the genome has repeat sequences (Alu and LINE) that are methylated in the normal state [44]. The role of these repeat sequences has not been incorporated into the outcomes of most of the epidemiology studies conducted thus far. A systematic study of the these sequences and their association should be conducted. These sequences are prone to integration by infectious agents and may contribute to the development of cancer.

In conclusion, there is a need to translate curiosity-driven basic research into patient-care-focused clinical research *via *epigenomics.

## Figures and Tables

**Table 1.** Samples Suitable for EWAS

Sample	Comment	Reference
Brocheoalveolar lavage fluid	Methylation analysis	[31]
Buccal cells	Methylation analysis	[45]
Cerebrospinal fluid		[31]
Ductal lavage fluid	Methylation analysis	[31]
Plasma	Methylation analysis	[46]
Saliva	Methylation analysis	[44]
Serum	Methylation analysis	[31, 46]
Stool	Methylation analysis	[46]
Sputum	Methylation analysis	[46]
Tissues	Methylation, histone, and miRNA analyses	[31, 46, 47]
Urine	Methylation analysis	[44, 46]

## ABBREVIATIONS

DNMT: = DNA Methyl Transferase
GWAS: = Genome-Wide Association Studies
EWAS: = Epigenome-Wide Association Studies
HAT: = Histone Acetyl Transferase
HDAC: = Histone Deacetylase
SAHA: = Suberoylanilide Hydroxamic Acid
SOCS: = Suppressors of Cytokine Signaling
